# Decreased Aortic Elasticity in Noncompaction Cardiomyopathy Compared to Dilated Cardiomyopathy

**DOI:** 10.3390/jcdd12080303

**Published:** 2025-08-11

**Authors:** Martijn Tukker, Sharida Mohamedhoesein, Emrah Kaya, Arend F. L. Schinkel, Kadir Caliskan

**Affiliations:** 1 Department of Cardiology, Office RG-431, Thoraxcenter, Cardiovascular Institute, ErasmusMC University Medical Center, Dr. Molewaterplein 40, 3015 GD Rotterdam, The Netherlands; 2 Department of Cardiology, Reinier de Graaf Hospital, Reinier de Graafweg 5, 2625 AD Delft, The Netherlands

**Keywords:** noncompaction cardiomyopathy, dilated cardiomyopathy, aortic disease, hypertrabeculation, aortic stiffness

## Abstract

Abnormal aortic elasticity serves as a marker for cardiovascular mortality and has a negative impact on the left ventricular (LV) afterload. Noncompaction cardiomyopathy (NCCM) is characterized by hypertrabeculation of the LV endomyocardial wall, with an underdeveloped endocardial helix. This may result in absence of LV twist, disturbed aortic elasticity, LV dysfunction, and ultimately premature heart failure (HF). This study compared the aortic stiffness and clinical outcome in patients with NCCM to that of a control group with dilated cardiomyopathy (DCM). Sixty NCCM patients, matched by age and sex, were compared with 60 DCM controls. Transthoracic echocardiography was performed to measure the systolic (SD) and diastolic diameters (DD) of the ascending aorta. These measurements, along with systolic (SBP) and diastolic blood pressure (DBP), were utilized to calculate the aortic stiffness index defined as ln(SBP/DBP)/[(SD-DD)/DD]. This index was then compared to clinical features and outcome. The mean age was 49 ± 16 years (55% males) in the NCCM group and 49 ± 16 years (55% male) in the DCM group. Aortic stiffness index (ASI) was significantly higher in the NCCM group than in the DCM group (7.0 [5.8–10.2] vs. 6.2 [4.8–7.7], *p* = 0.011). This difference remained statistically significant after adjustment for established risk factors associated with aortic stiffness (β = 1.771; 95% CI [0.253–3.289], *p* = 0.023). Patients with NCCM demonstrated increased aortic stiffness when compared to those with DCM, which may reflect the underlying pathophysiological processes. Additional research is necessary to evaluate the impact of aortic stiffness on the advancement of LV dysfunction, the onset of heart failure, and long-term outcomes.

## 1. Introduction

Abnormal aortic elasticity, also known as aortic stiffness, is a recognized indicator of cardiovascular mortality [1,2]. Aortic stiffness refers to the impaired ability of the aorta to expand and recoil in response to changes in pressure. Age, diabetes mellitus, heart failure (HF), hypertension, and atherosclerosis have been identified as factors associated with increased aortic stiffness [1,3]. The aortic stiffness index (ASI) is a method used to quantify aortic elasticity [4]. Previous studies have demonstrated that increased aortic stiffness is also linked to reduced functional capacity, HF rehospitalization, and myocardial fibrosis in patients with cardiomyopathies [2,5,6].

Noncompaction cardiomyopathy (NCCM) is characterized by the presence of hypertrabeculation of the left ventricular (LV) endomyocardial layer, a thin epicardial myocardial layer, and a diminished LV twist [7,8]. These anatomical features can result in a range of clinical manifestations, including LV dysfunction, which can progress to HF, (supra)ventricular arrhythmias, sudden cardiac death (SCD), and thromboembolisms [9,10]. Both dilated cardiomyopathy (DCM) and NCCM are characterized by LV dysfunction and the development of chronic heart failure. DCM, however, differs from NCCM regarding normal trabeculation and LV twist [7].

The aim of this study was to assess the aortic stiffness and clinical outcomes in patients with NCCM patients in comparison to a control group suffering from DCM.

## 2. Materials and Methods

### 2.1. Study Population

This retrospective single-center study included patients diagnosed with NCCM, matched by age and sex, and compared them to DCM patients. All patients were enrolled in the Rijnmond Heart Failure/Cardiomyopathy Registry at the Erasmus University Medical Center in the Netherlands between February 2014 and January 2021. The study was approved by the local ethics committee, and all patients provided informed consent.

Patients diagnosed with NCCM exhibited LV wall hypertrabeculation, with the diagnosis established when the ratio of the noncompact endocardial layer to the compacted epicardial layer, as observed in the parasternal short-axis view at the end of systole, exceeded 2.0 [11]. The diagnostic criteria for DCM were determined based on LV dilatation and global or regional systolic dysfunction that could not be explained solely by abnormal loading conditions or coronary artery disease [12].

Patients aged 18 years or older with a confirmed diagnosis of NCCM or DCM and the availability of high-quality transthoracic echocardiography (TTE) images were eligible for case–control matching. Patients were excluded if they did not have high-quality TTE images available or had undergone heart transplantation or left ventricular assist device (LVAD) implantation prior to collecting the used TTE images. Subsequently, eligible NCCM patients were matched with DCM patients at a 1:1 ratio on the basis of sex and age within a 5-year range. A total of 60 patients with satisfactory image quality were included in the study for whom ASI measurements were feasible.

### 2.2. Measurement of Blood Pressure and Aortic Diameters

**Figure 1:**
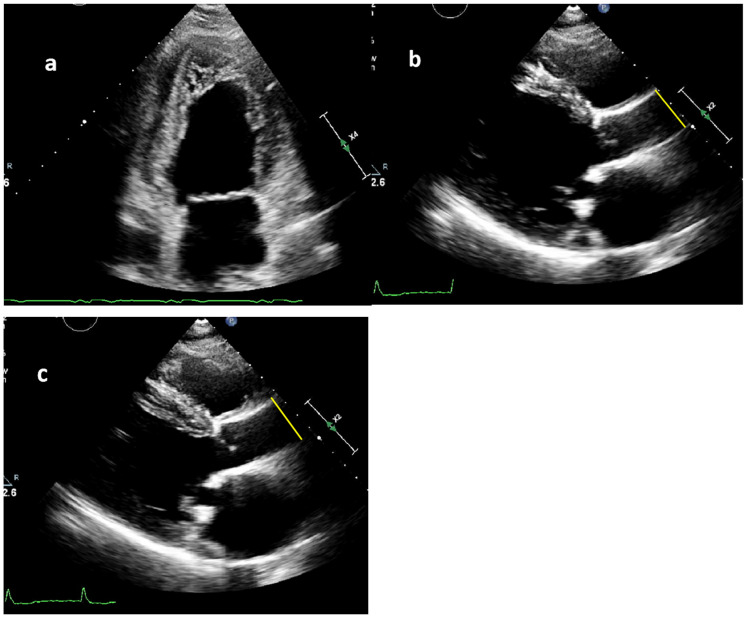
(**a**–**c**): Measuring technique of the systolic and diastolic ascending aortic diameters. (**a**) The two-chamber view of the left ventricle and left atrium in a noncompaction cardiomyopathy patient. (**b**,**c**)The transthoracic echocardiographic images of a 51-year-old male with dilated cardiomyopathy (DCM). He could only walk 200 m at presentation. (**b**) The yellow line presents the measurement of the diastolic aortic diameter (26 mm). (**c**) The yellow line presents the measurement of the systolic aortic diameter (29 mm).

Systolic and diastolic blood pressure (SBP and DBP) were measured using an automatic sphygmomanometer with the patient in a seated position. The TTE images, obtained following enrollment in the registry, were analyzed to measure the systolic and diastolic ascending aortic diameters (SD and DD). Measurements were conducted in the two-dimensional parasternal long-axis view (PLAX), at a distance of 4 cm from the aortic valve, in a strictly perpendicular plane from leading edge to leading edge [13]. For the calculation of maximal aortic elasticity, SD was assessed during the maximum anterior movement of the aortic wall. The maximum relaxation of the ascending aorta was assessed at the R peak of the QRS complex, which was used as a reference point for determining DD (see Figure 1).

### 2.3. Aortic Stiffness Index

Aortic elasticity was indexed using the blood pressure and aortic diameter values, employing the aortic stiffness index (ASI) method [4]. The ASI is calculated using the following formula: ASI = ln(SBP/DBP)/[(SD-DD)/DD].

### 2.4. Statistics

The normality of continuous variables was evaluated using the Shapiro–Wilk test. For variables with a normal distribution, the mean ± standard deviation was used to present the data. Conversely, for variables with a non-normal distribution, the median and interquartile range were used. Comparisons of continuous variables were performed using either the Student’s *t*-test or the Wilcoxon–Mann–Whitney test. Categorical variables were expressed as frequencies (n) and percentages (%), and comparisons were made using the Chi-squared test or Fisher’s exact test. A linear regression model was constructed to adjust for potential confounding variables. To assess intra-observer agreement, a random sample of 25 NCCM patients and 25 DCM patients underwent repeated analysis. The initial result was blinded for the sample. To assess inter-observer agreement, a second observer (SM) analyzed the same sample. The degree of agreement between two measurements was determined as the mean of the differences +1.96 SD. A significance level of *p* < 0.05 was considered statistically significant. The data were analyzed using IBM SPSS Statistics 28.0.1.0 and R version 4.3.2.

## 3. Results

The baseline characteristics of these subjects are presented in Table 1 and Table 2. The mean age at aortic diameter measurement was 49 ± 15 years (55% male) in the NCCM group and 49 ± 16 years (55% male) in the DCM group. Diabetes was more prevalent in patients with DCM than in those with NCCM (23% vs. 2%; *p* = 0.006). The DCM group used significantly more medications compared to the NCCM group, including beta-blockers, ACE/ARB inhibitors, diuretics, and aldosterone receptor antagonists, along with a lower mean LV ejection fraction (LVEF) in DCM patients compared to NCCM patients: 34 ± 12% vs. 42 ± 11%; *p* = 0.004. No significant differences were observed in the incidence of ventricular arrhythmias between the two groups.

**Figure 2:**
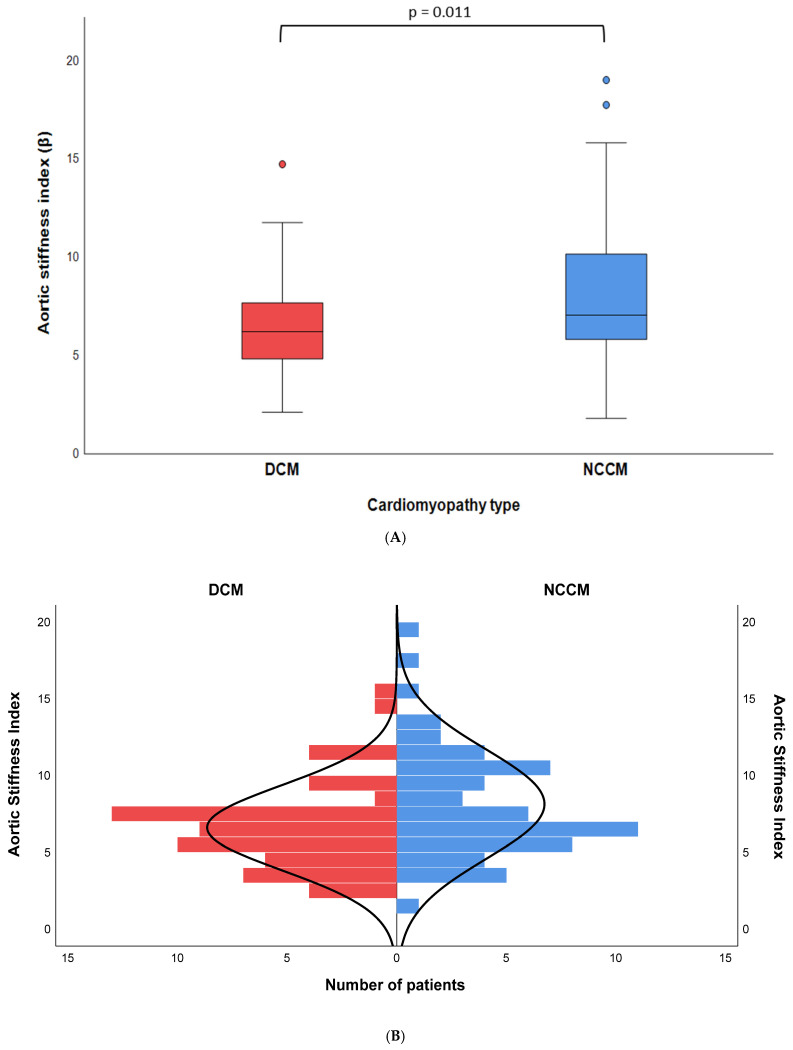
(**A**) A box plot showing the aortic stiffness index in NCCM and DCM patients. (**B**) A mirrored histogram showing the aortic stiffness index distribution in NCCM and DCM patients.

The median ASI was 7.0 [5.8–10.2] in the NCCM group, in comparison to 6.2 [4.8–7.7] in the control group. The observed difference between the two groups was statistically significant (*p* = 0.011) (Figure 2A,B). No statistically significant differences were observed in the systolic and diastolic ascending aortic diameters between the NCCM and DCM groups. However, the NCCM group exhibited a significantly higher mean systolic and diastolic blood pressure than the control group (128 ± 16 vs. 117 ± 18; *p* = 0.001 and 77 ± 9 vs. 70 ± 10; *p* = 0.001, respectively). Moreover, comparisons based on sex demonstrated that males in the NCCM group had elevated ASI scores compared to those observed in males DCM patients (7.2 [6.5–10.9] vs. 5.8 [4.1—7.7]; *p* = 0.005). No differences in ASI were found between the two groups in female patients.

The median follow-up period was 24 months [15–40] in the NCCM group and 37 months [18–78] in the DCM group (Table S1). During the follow-up period, 1 patient in the NCCM group died compared to 11 patients in the DCM group (*p* = 0.006). However, there was no significant difference in the incidence of cardiovascular mortality between the two groups (2% vs. 10%, *p* = 0.815). Seven patients with DCM (12%) experienced ventricular arrhythmias, leading to six of them receiving an ICD-appropriate shock. Additionally, significantly more DCM patients underwent heart transplantation or LVAD implantation during the follow-up period in comparison to NCCM patients (2% vs. 17%; *p* = 0.011).

The results of the linear regression model indicated that NCCM was associated with a significantly higher ASI when corrected for age and sex (β = 1.714; 95% CI [0.651–2.778], *p* = 0.002) (Table 3). This remained significant after adjusting for clinical characteristics, including age at echocardiography, sex, hypertension, diabetes mellitus, hypercholesterolemia, LVEF, and HF (β = 1.667; 95% CI [0.220–3.114], *p* = 0.025). Model 3 was adjusted for clinical characteristics and HF medication (beta-blockers, ACE-inhibitors/angiotensin receptor blockers, diuretics, aldosterone antagonists). Following correction for these variables, NCCM was found to be significantly associated with a higher ASI (β = 1.771; 95% CI [0.253–3.289], *p* = 0.023).

The intra- and inter-observer agreement for aortic measurement was evaluated in a randomly selected subset of 25 patients with NCCM and 25 patients with DCM in both systolic and diastolic views (Figure S1). The inter-observer variability demonstrated a mean difference of −0.04 ± 1.81 mm for systolic diameters and −0.72 ± 1.81 mm for diastolic aorta diameter measurements. The intra-observer variabilities demonstrated a mean difference of 1.28 ± 1.34 mm for systolic diameters and 0.79 ± 1.40 mm for diastolic aorta diameter measurements. Subsequently, the variability of the ASI was analyzed by implementing the aforementioned measurements with the specified formula. The mean difference for ASI inter-observer variability was −3.16 ± 4.68, while the mean difference for ASI intra-observer variability was −1.01 ± 3.27. Finally, observer variability was measured for the difference between systolic and diastolic diameters (SD-DD). The mean difference for SD-DD inter-observer variability was 0.68 ± 1.17 mm, while the mean difference for ASI intra-observer variability was 0.48 ± 1.11 mm.

## 4. Discussion

The results of this study indicate that the NCCM group had a higher median ASI compared to the DCM group (7.0 [5.8–10.2] vs. 6.2 [4.8–7.7], *p* = 0.011). This association remained statistically significant even after adjustment for potential confounders (β = 1.771; 95% CI [0.253–3.289], *p* = 0.023).

Aortic stiffness has been identified as a predictor of cardiovascular and all-cause mortality [14]. The existing literature on DCM and aortic stiffness is more extensive than that on NCCM and aortic stiffness. Vizzardi et al. observed a mean ASI of 15.6 ± 14.5 in patients with DCM and HF. Furthermore, aortic stiffness has been identified as a factor that negatively affects functional capacity in DCM patients with HF [6]. Patrianakos identified plasma BNP levels as an independent predictor of aortic stiffness in DCM patients with HF [15]. Further investigation into neurohormonal differences by Bonapace et al. revealed that higher serum PIIINP levels, a marker of extracellular matrix turnover, were associated with a stiffer aorta [16]. This suggests that ECM turnover may influence ASI, which may explain why HF patients have higher aortic stiffness. Sciatti et al. focused on ASI as a predictor in DCM patients [2]. The mean ASI was 6.7 ± 0.8, and ASI was identified as a predictor of HF rehospitalization and mortality. The existing literature on aortic stiffness in NCCM is limited. In a study by Nemes et al., NCCM patients were compared with healthy controls, and a higher ASI was observed in the NCCM cohort (8.3 ± 5.2 vs. 3.5 ± 1.1, *p* < 0.001) [17]. The study suggested that the observed difference was caused by neurohormonal differences.

Increased aortic stiffness has also been observed in patients with heart failure with preserved ejection fraction (HFpEF) [18]. Since aortic stiffness increases LV afterload, it can contribute to the development of diastolic dysfunction, a key precursor of HFpEF. A similar pattern is seen in centenarians, who experience age-related reductions in vascular compliance, placing them at higher risk for diastolic dysfunction [19]. Therefore, aortic stiffness holds potential as a prognostic marker for NCCM patients at risk for the development of HF. This study is the first to investigate aortic stiffness in NCCM patients compared to DCM patients. DCM patients present with premature LV dysfunction and HF. However, in contrast to NCCM patients, they do not have abnormal LV twist or hypertrabeculation of the LV myocardial wall [7]. The NCCM cohort had a higher ASI, although the DCM cohort had a higher prevalence of diabetes, use of HF medications, and adverse clinical outcomes. These findings may indicate that LV twist and/or hypertrabeculation may influence ASI. An examination of the absolute difference in median ASI between the two cohorts shows that both are significantly higher than the median ASI of the healthy cohort reported by Nemes et al. [17]. However, given that previous studies have reported a mean ASIs of up to 15.6, the observed difference between DCM and NCCM and the potential impact of hypertrabeculation and reduced LV twist on ASI remain relatively modest.

Clinical outcomes were more frequent in the DCM group compared to the NCCM group. This may be partially explained by the longer follow-up period in DCM patients compared to that in NCCM patients (median 37 months vs. 24 months), the lower baseline LVEF, and the higher prevalence of diabetes in the DCM group. Additionally, the difference in ASI between the groups may not have been substantial enough to observe a significant impact of the higher ASI in NCCM on clinical outcomes during the follow-up period.

### Limitations

This study has several potential limitations. The accuracy of the aortic diameters depends on the quality of the TTE images. This is why of the original 109 patients, only 60 NCCM patients remained for matching. In addition, the measurement of systolic and diastolic ascending aortic diameters in PLAX view was conducted using the leading edge method in absence of M-mode. Although M-mode measurements have been used in other studies, they were not available in this study due to a lack of data. It is important to note that M-mode, being a two-dimensional technique, has the potential for diagonal tracing of the aorta, which may result in inaccurate measurements. Therefore, the use of an alternative method in this study may provide more reliable measurements. Despite matching the NCCM and DCM groups for age and sex, baseline characteristics differed significantly in terms of diabetes prevalence and the presence of advanced HF. Although these variables were adjusted for in the multivariable regression analysis, the possibility of residual confounding cannot be excluded. ASI was measured using a standardized method, albeit with inter- and intra-observer variability that exceeded the observed median ASI difference between DCM and NCCM groups.

## 5. Conclusions

The results of this study suggest that patients with NCCM have increased aortic stiffness compared to patients with DCM. While the absolute difference between NCCM and DCM is relatively modest, these findings may reflect differences in the underlying pathophysiological mechanisms, which can be used to generate hypotheses and warrant further investigation. Further studies are needed to evaluate the impact of aortic stiffness on the progression of LV dysfunction, the development of heart failure, and long-term prognosis.

## Figures and Tables

**Table 1:** Baseline characteristics of the study populations.

Variable	DCM (n = 60)	NCCM (n = 60)	*p*-Value
**Demographics**			
Age at echo, years	49 ± 16	49 ± 16	0.870
Age at presentation, years	37 ± 14	40 ± 16	0.286
Males	33 (55)	33 (55)	
**Comorbidities**			
Hypertension	17 (28)	13 (22)	0.527
Diabetes	14 (23)	1 (2)	0.001
Hypercholesterolemia	4 (7)	6 (10)	0.741
Coronary artery disease	8 (13)	5 (8)	0.557
Ventricular arrhythmia	11 (18)	10 (17)	1.000
Heart failure hospitalization	15 (25)	11 (18)	0.506
Stroke	2 (3)	3 (5)	1.00
CAD	0 (0)	1 (2)	1.00
Stroke	2 (3)	3 (5)	1.00
**Primary presentation**			
Heart failure	24 (40)	18 (30)	0.339
**Physical Examination**			
Height, m	1.76 ± 10	1.76 ± 10	0.785
Weight, kg	82 ± 18	81 ± 17	0.626
Systolic BP, mmHg	117 ± 18	128 ± 16	0.001
Diastolic BP, mmHg	70 ± 10	77 ± 9	0.001
**Medication**			
Beta-receptor antagonist	57 (95)	44 (73)	0.003
ACE-inhibitor/ARB	54 (90)	39 (65)	0.002
Diuretics	44 (73)	14 (23)	<0.001
Aldosterone receptor antagonist	38 (63)	13 (22)	<0.001
Devices,			
ICD implantation	41 (70)	28 (48)	0.025
Appropriate therapy	8 (20)	5 (19)	1.000
Inappropriate therapy	2 (5)	3 (11)	0.628

Continuous variables are summarized by mean ± SD or median (IQR), categorical variables are described as: n (%). ACE = angiotensin converting enzyme; ARB = angiotensin receptor blocker; BP = blood pressure; CAD = coronary artery disease; ICD = implantable cardioverter–defibrillator.

**Table 2:** Transthoracic echocardiogram findings in NCCM and DCM patients.

Variable	DCM (n = 60)	NCCM (n = 60)	*p*-Value
LA diameter, mm	41 ± 8	39 ± 7	0.241
LVED diameter, mm	60 ± 11	58 ± 9	0.427
LVES diameter, mm	47 ± 16	44 ± 13	0.397
LVEF, %	34 ± 12	42 ± 11	0.004
Aortic regurgitations	3 (7)	6 (11)	0.623
Systolic ascending aortic diameter, mm	30 ± 5	31 ± 5	0.883
Diastolic ascending aortic diameter, mm	28 ± 4	29 ± 5	0.483
Aortic stiffness index ^#^	6.2 [4.8–7.7]	7.0 [5.8–10.2]	0.011

Continuous variables are summarized by mean ± SD or median (IQR), categorical variables are described as n (%). ASI = aortic stiffness index; LA = left atrium; LVED = left ventricular end diastole; LVEF = left ventricular ejection fraction; LVES = left ventricular end systole. ^#^ Aortic stiffness index was calculated using the following formula: ln[(SBP/DBP)/(SD-DD)/DD].

**Table 3:** Linear regression for differences in aortic stiffness index between NCCM and DCM patients.

	Model 1 Corrected for Age and Sex			Model 2 Corrected for Clinical Characteristics			Model 3 Corrected for Clinical Characteristics and Heart Failure Medication		
Variables:	β	CI 95%	*p*-Value	β	CI 95%	*p*-Value	β	CI 95%	*p*-Value
Noncompaction CMP	1.714	0.651–2.778	0.002	1.667	0.220–3.114	0.025	1.771	0.253–3.289	0.023

Model 1: Corrected for age at echocardiography date and sex. Model 2: Corrected for age at echocardiography date, sex, hypertension, diabetes mellitus, hypercholesterolemia, left ventricular ejection fraction, heart failure. Model 3: Corrected for age at echocardiography date, sex, hypertension, diabetes mellitus, hypercholesterolemia, left ventricular ejection fraction, heart failure, taking at least 1 heart failure medication (beta-blockers, ACE-inhibitors/angiotensin receptor blockers, diuretics, aldosterone antagonists); CMP = cardiomyopathy.

## Data Availability

The datasets presented in this article are not readily available because due to technical limitations. Requests to access the datasets should be directed to dr.kcaliskan@outlook.com.

## Supplementary Materials

The following supporting information can be downloaded at: https://www.mdpi.com/article/10.3390/jcdd12080303/s1, Figure S1a: Inter-observer variability in aortic systolic measurements; Figure S1b: Inter-observer variability in aortic diastolic measurements; Figure S1c: Intra-observer variability in aortic diastolic measurements; Figure S1d: Intra-observer variability in aortic systolic measurements; Figure S1e: Inter-observer variability in aortic stiffness index; Figure S1f: Intra-observer variability in aortic stiffness index; Figure S1g: Inter-observer variability in systolic minus diastolic aortic diameter measurements; Figure S1h: Intra-observer variability in systolic minus diastolic aortic diameter measurements; Table S1: Clinical outcome differences between NCCM and DCM at follow-up.

## Abbreviations

The following abbreviations are used in this manuscript:

ASI	Aortic stiffness index
DCM	Dilated cardiomyopathy
HF	Heart failure
ICD	Implantable cardioverter–defibrillator
LVAD	Left ventricular assist device
LV	Left ventricle
LVEF	Left ventricular ejection fraction
NCCM	Noncompaction cardiomyopathy
PLAX	Parasternal long-axis view
SCD	Sudden cardiac death
TTE	Transthoracic echocardiography
