# Supplementary material for: Decreased Aortic Elasticity in Noncompaction Cardiomyopathy Compared to Dilated Cardiomyopathy
Source: J Cardiovasc Dev Dis. 2025 Aug 11;12(8):303. doi: 10.3390/jcdd12080303 (PMC12386488; doi:10.3390/jcdd12080303)
Supplement: Supplementary file 1 [file jcdd-12-00303-s001.zip › jcdd-3672121-supplementary.pdf]

Supplemental material

Supplemental Figures

Figure S1a. Inter-observer variability in aortic systolic measurements.

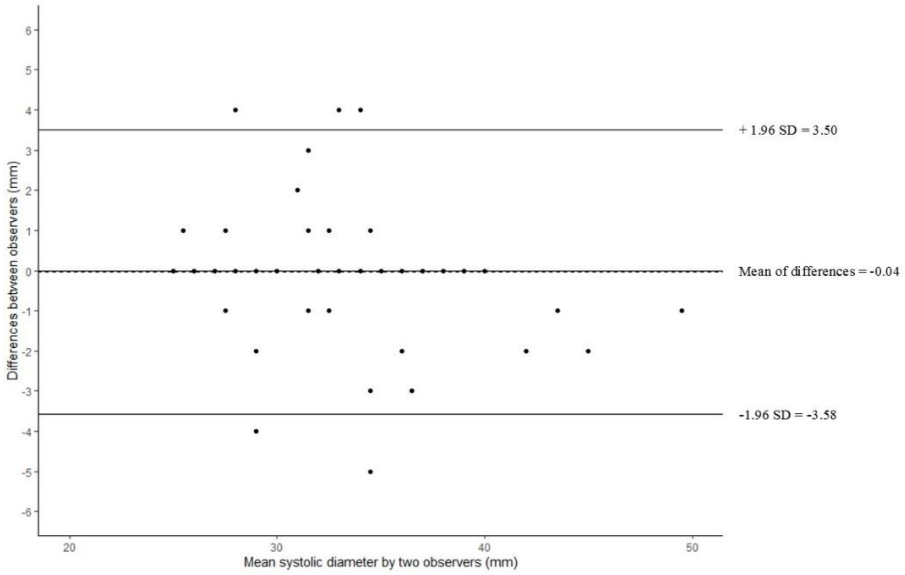

Figure S1b. Inter-observer variability in aortic diastolic measurements.

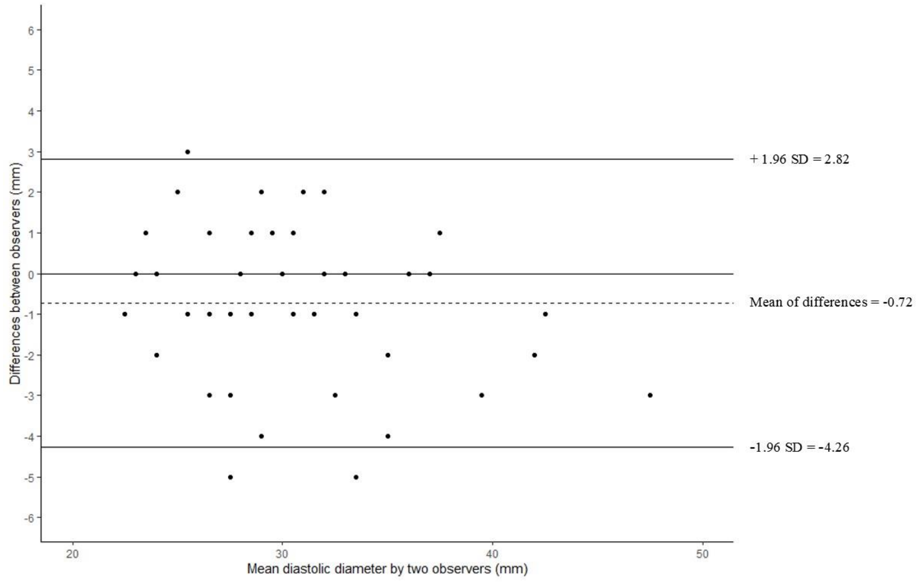

Figure S1c. intra-observer variability in aortic diastolic measurements.

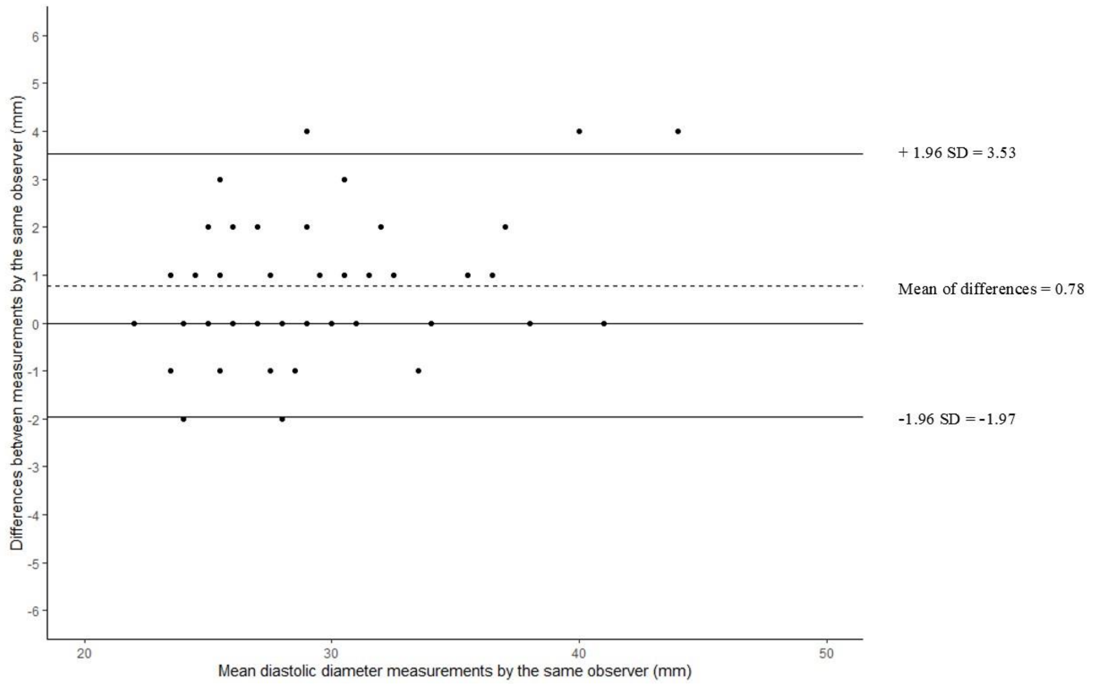

Figure S1d. intra-observer variability in aortic systolic measurements.

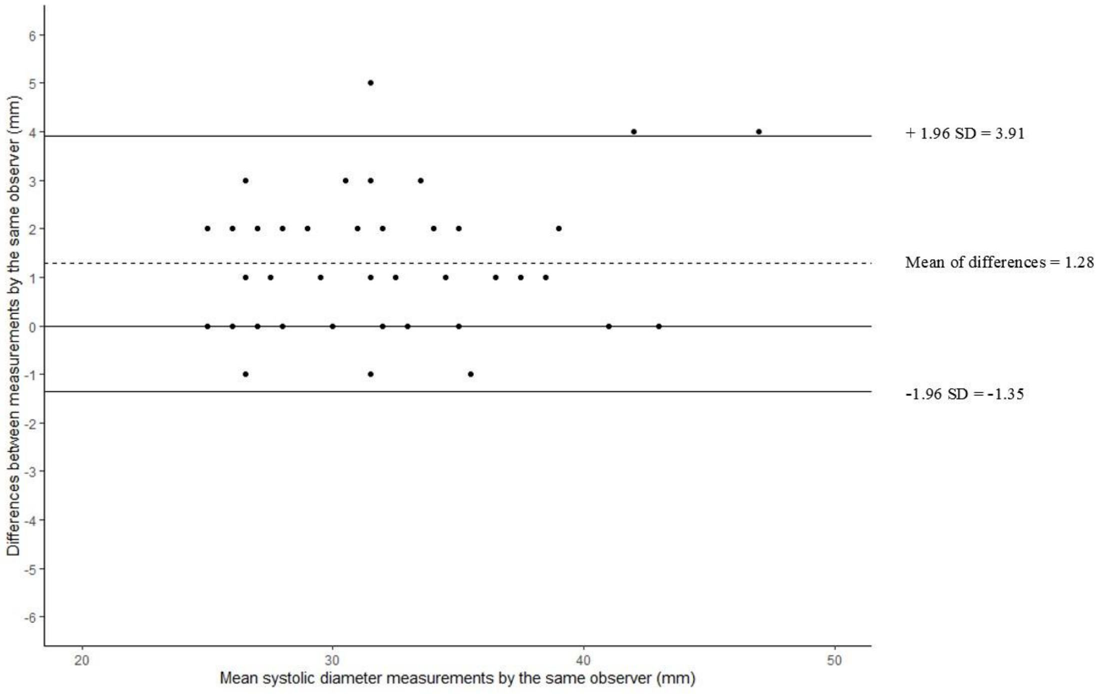

Figure S1e Inter-observer variability in aortic stiffness index.

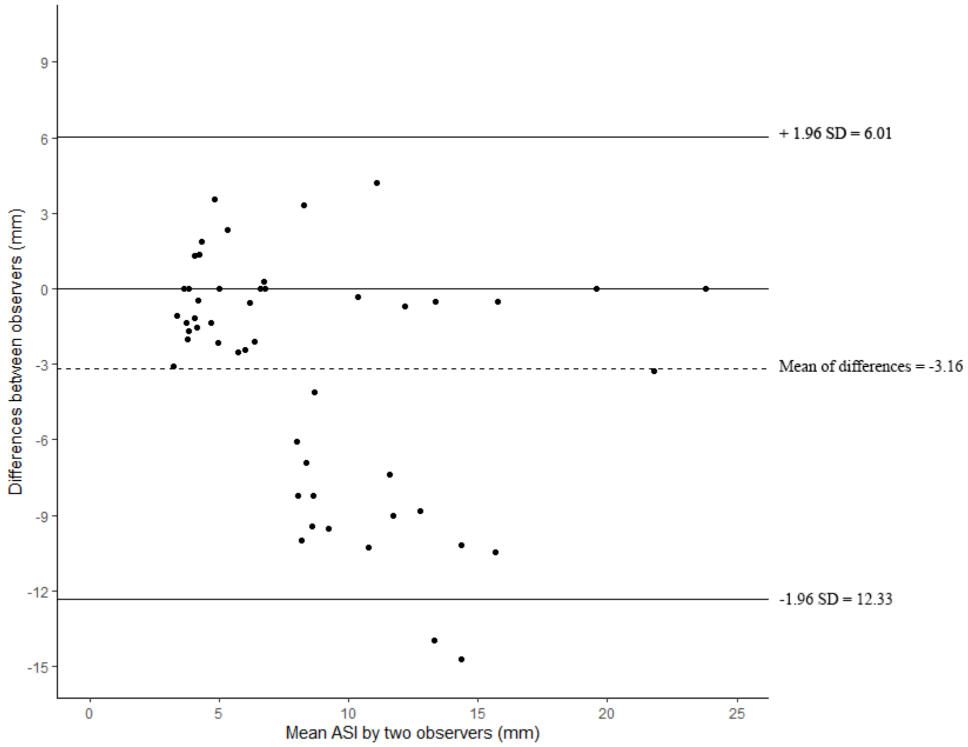

Figure S1f Intra-observer variability in aortic stiffness index.

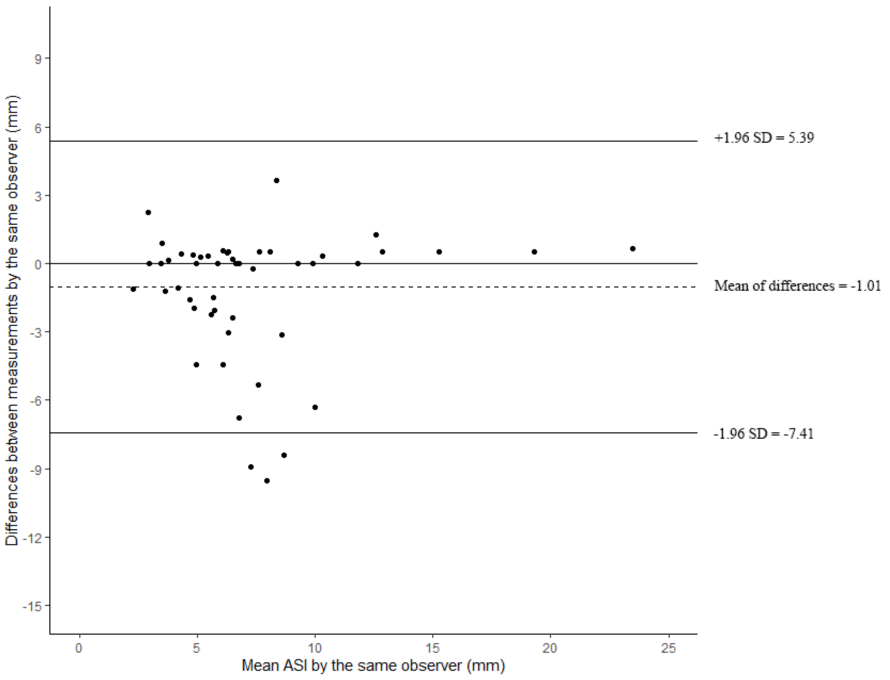

Figure S1g. Inter-observer variability in systolic minus diastolic aortic diameter measurements.

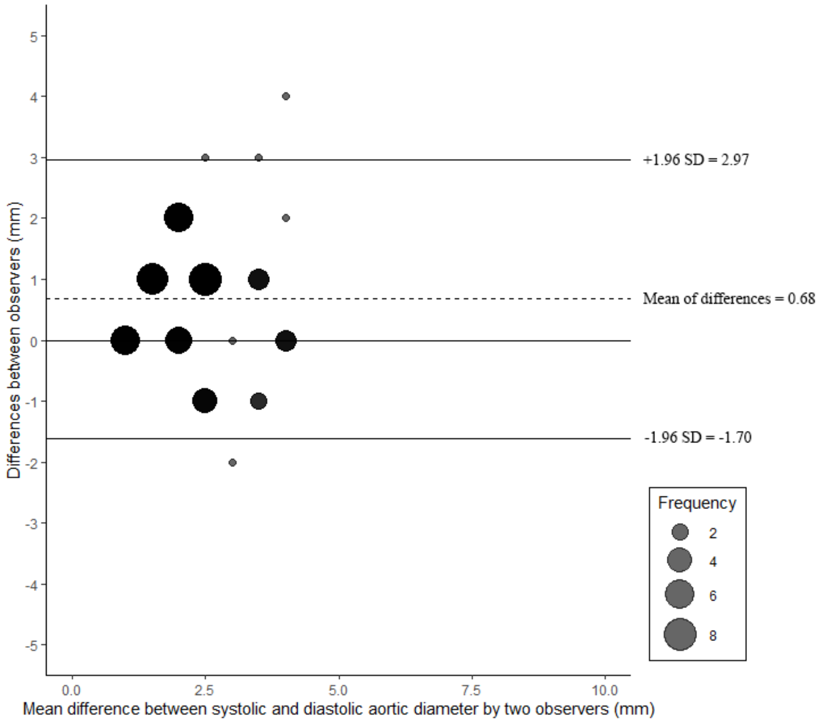

Figure S1h. Intra-observer variability in systolic minus diastolic aortic diameter measurements.

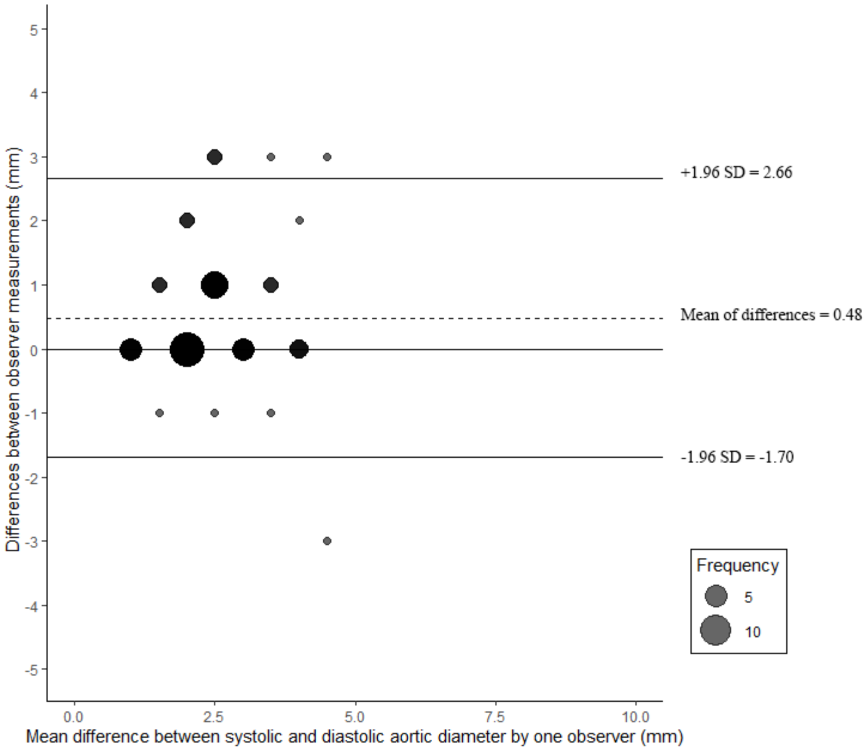

Supplemental Table

Table S1. Clinical outcomes differences between NCCM and DCM at follow-up.

Variable	DCM	NCCM	p-value
Follow-up, months	37 [18 - 78]	24 [15 - 40]	0.019
**Devices, n (%)**			
ICD appropriate therapy	6 (15)	0 (0)	0.036
ICD inappropriate therapy	0 (0)	0 (0)	
**Outcomes, n(%)**			
Ventricular arrhythmia	7 (12)	0 (0)	0.019
Heart failure hospitalization	1 (2)	1 (2)	1.000
Stroke	0 (0)	0 (0)	
CAD	0 (0)	0 (0)	
Heart transplantation	10 (17)	1 (2)	0.011
Cardiovascular death	6 (10)	1 (2)	0.815

CAD= coronary artery disease; ICD = implantable cardioverter-defibrillator; LVAD = left ventricular assist device.
